# Transmission blocking activity of *Azadirachta indica* and *Guiera senegalensis* extracts on the sporogonic development of *Plasmodium falciparum* field isolates in *Anopheles coluzzii* mosquitoes

**DOI:** 10.1186/1756-3305-7-185

**Published:** 2014-04-15

**Authors:** Rakiswendé S Yerbanga, Leonardo Lucantoni, Robert K Ouédraogo, Dari F Da, Franck A Yao, Koudraogo B Yaméogo, Thomas S Churcher, Giulio Lupidi, Orazio Taglialatela-Scafati, Louis Clément Gouagna, Anna Cohuet, George K Christophides, Jean Bosco Ouédraogo, Annette Habluetzel

**Affiliations:** 1 Institut de Recherche en Sciences de la Santé, 01 BP545 Bobo Dioulasso, Burkina Faso; 2 Scuola di Scienze del Farmaco e dei Prodotti della Salute, Università di Camerino, 62032 Camerino, MC, Italy; 3 Department of Infectious Disease Epidemiology, Imperial College London, London W2 1PG, United Kingdom; 4 Dipartimento di Farmacia, Università di Napoli “Federico II”, via D. Montesano, 49, I-80131 Napoli, Italy; 5 Institut de Recherche pour le Développement, Unité MIVEGEC (IRD 224- CNRS 5290-UM1-UM2), BP 64501 Montpellier Cedex 5 34394, France; 6 Department of Life Sciences, Imperial College London, London, United Kingdom

**Keywords:** *Plasmodium falciparum*, Gametocytes, Sporogonic stages, Plant extracts, Transmission-blocking drugs

## Abstract

**Background:**

Targeting the stages of the malaria parasites responsible for transmission from the human host to the mosquito vector is a key pharmacological strategy for malaria control. Research efforts to identify compounds that are active against these stages have significantly increased in recent years. However, at present, only two drugs are available, namely primaquine and artesunate, which reportedly act on late stage gametocytes.

**Methods:**

In this study, we assessed the antiplasmodial effects of 5 extracts obtained from the neem tree *Azadirachta indica* and *Guiera senegalensis* against the early vector stages of *Plasmodium falciparum,* using field isolates. In an *ex vivo* assay gametocytaemic blood was supplemented with the plant extracts and offered to *Anopheles coluzzii* females by membrane feeding. Transmission blocking activity was evaluated by assessing oocyst prevalence and density on the mosquito midguts.

**Results:**

Initial screening of the 5 plant extracts at 250 ppm revealed transmission blocking activity in two neem preparations. Up to a concentration of 70 ppm the commercial extract NeemAzal® completely blocked transmission and at 60 ppm mosquitoes of 4 out of 5 replicate groups remained uninfected. Mosquitoes fed on the ethyl acetate phase of neem leaves at 250 ppm showed a reduction in oocyst prevalence of 59.0% (CI_95_ 12.0 - 79.0; *p* < 10^-4^) and in oocyst density of 90.5% (CI_95_ 86.0 - 93.5; *p* < 10^-4^ ), while the ethanol extract from the same plant part did not exhibit any activity. No evidence of transmission blocking activity was found using *G. senegalensis* ethyl acetate extract from stem galls.

**Conclusions:**

The results of this study highlight the potential of antimalarial plants for the discovery of novel transmission blocking molecules, and open up the potential of developing standardized transmission blocking herbal formulations as malaria control tools to complement currently used antimalarial drugs and combination treatments.

## Background

Thanks to the advancements of knowledge and to a significant expansion of financial resources supporting malaria programmes, the burden of the disease has been significantly reduced over the last decade. An estimated 274 million cases and 1.1 million deaths have been averted and 50 countries with ongoing malaria transmission are on track to reduce the incidence of malaria cases by 75% by 2015 [1]. However, since current and prospective international disbursements for malaria control reach not even half of the estimated yearly US$ 5.1 billion required to achieve universal coverage of malaria interventions [1], it is highly uncertain whether the international targets for reducing malaria cases and deaths can be attained in all stricken countries.

Moreover the progress achieved remains under threat from the development of artemisinin resistant parasites, which have already been detected in 4 countries of the South East Asia Region [1]. The likelihood of resistance emergence is increased with the spread of poor-quality drugs. A recent review of studies conducted in 7 Asian and 21 sub-Saharan African countries unveiled an alarming occurrence of falsified, substandard and degraded anti-malarial drugs with about one third of the drug samples tested failing chemical analysis by pharmacopeia standards [2].

This scenario calls for the acceleration of the discovery of novel antiplasmodial molecules and the design and development of new combination drugs tailored to the pharmacological needs of malaria control (i.e. to cure and prevent the disease in individuals and control/eliminate transmission). Combination drugs including molecules effective against transmissible stages of the parasite have a key role to play both as a resistance containment strategy and to equip countries entering the malaria elimination phase with the required tools. Current ACTs based on artemisinin compounds which are active on asexual blood stages as well as gametocytes have been shown to have a beneficial impact on transmission [3-5] despite being only partially effective [6]. The parasite’s success in escaping elimination by artemisinin based drugs is associated with the molecules limited efficacy against the mature sexual stages in the vertebrate host [7], the short half-life of artemisinins and their active metabolites [8] and the long persistence (2–3 weeks) of mature gametocytes. Indeed, mature gametocytes are difficult targets for drug attack, which makes it a challenge to devise drugs that interfere with its biochemical pathways. As illustrated by early studies of RE Sinden [9] immature gametocytes are sensitive to inhibitors of DNA, RNA and protein synthesis, including antimalarials, but later stages to a minor extent. A treatment scheme with two different gametocytocidal molecules can be a practical approach, as shown by the study of Shekalaghe and colleagues [6] in which they showed that the administration of primaquine after a treatment course of sulphadoxine/pyrimethamine and artesunate caused an additional decrease in gametocyte densities to less than 0.1 per microliter of blood, a density at which the probability of mosquito infection is drastically reduced.

From a parasitologist’s perspective, there are several arguments to support focusing on the early sporogonic stages developing in the midgut lumen of the mosquito host. First, transition from the vertebrate to the phylogenetically completely different mosquito host is an extremely critical phase in the parasite’s life cycle. This may well explain why even without the introduction of an intervention the yield of the sexual process is so low. In rodent plasmodia it has been estimated that, from 500 macrogametocytes counted in the vertebrate host, only one goes through the whole process and develops into an oocyst on the midgut wall [10]. After blood ingestion by the mosquito, the sexual parasite stages undergo profound biological transformation and maturation within a few hours, involving numerous cellular and biochemical processes that offer plentiful potential drug targets [11]. Also, during this phase the parasites are extracellular, i.e. they lack protection by a host cell membrane, which facilitates drug interference with the parasite.

On this background it is not surprising that a large number of schizonticidal drugs designed to target asexual erythrocytic forms were found to display activity, and in some cases multi-stage effects, against the various transmissible Plasmodium stages [12]. For example, endoperoxides, such as OZ439, a synthetic molecule currently under clinical phase IIa trials, is a strong inhibitor of gametocyte maturation, gamete formation and has an impact on sporogony; lumefantrine and NPC-1161B, a new 8-aminoquinoline, also inhibits sporogony [12].

Learning the lesson from ACTs, i.e. the successful development of highly effective artemisinin based combination drugs starting from the medicinal plant *Artemisia annua*, prompted us to explore plants frequently used as anti-malarial remedies for possible transmission blocking effects. Among the plant species investigated, extracts from *Azadirachta indica* and *Guiera senegalensis* revealed *in vivo* transmission blocking activity (*Plasmodium berghei, Anopheles stephensi, BALB/c mice)* and/or *in vitro* inhibitory effects on the early sporogonic development of *P. berghei*. The *Azadirachta indica* extract, NeemAzal®, tested *in vitro* at 6.5 μg/ml resulted in a 65.8% inhibition of early sporogonic stage development and a *Guiera senegalensis* ethylacetate fraction of galls tested at 50 ug/ml showed a 66% to 88% inhibitory activity (unpublished results). In particular, NeemAzal® a commercial (Trifolio-M GmbH, Lahnau, Germany) methanol extract from seed kernels of *A. indica* rich in Azadirachtin A, showed prominent effects, completely inhibiting mosquito infection when administered intraperitoneally at a dosage of 50 mg/kg to gametocytaemic mice [13,14].

The present study was undertaken to assess the activity of these plants on the transmissible stages of the human malaria parasite under field-like conditions. *P. falciparum* gametocytes from naturally infected humans and *Anopheles coluzzii* mosquitoes from colonies established in 2008 were used. Female mosquitoes were membrane fed with blood supplemented with plant extracts. In order to explore which compounds are likely to be responsible for transmission blocking activity, chemically characterized extracts prepared with various solvents (MeOH, EtOH, EtOAc) from different plant parts were tested.

## Methods

### Study area and recruitment of *P. falciparum* gametocyte carriers

The study was conducted in the Bobo Dioulasso area (Burkina Faso) from May to October 2011. Malaria transmission is hyperendemic in the area, with an estimated *P. falciparum* entomological inoculation rate of 300–500 infective bites per person per year. Parasite prevalence and densities are strongly seasonal, with the transmission season peaking in September and lasting from approximately June to October. Gametocyte-positive blood samples for the direct membrane feeding assay (DMFA) were obtained from children, residents of the Dandé, Soumousso and Bama villages, situated at 40–55 km distance from Bobo-Dioulasso town.

A total of 2160 children aged 5 – 11 years participated at 18 screening events organized from May to October 2011. For each event, groups of 120 children were invited to present at the village health center early in the morning. Every child was clinically examined for the presence of chronic diseases, acute infections other than malaria and signs of severe malaria. Finger-prick blood was collected and used for the preparation of thick smears. Information on anti-malarial drugs taken during the preceding 2 weeks as well as presence of hypersensitivity to anti-malarial drugs was recorded.

Thick smears were stained with Giemsa and examined in the laboratory on the same day. On each slide 100 fields were screened for the presence of *Plasmodium* parasites. Asexual parasite and gametocyte numbers were calculated per 200 and 1000 leukocytes respectively. Gametocytemia and parasitemia were then expressed as number of gametocytes or total number of parasites per microliter (μL) of blood, assuming leukocyte counts of 8000/μL of blood.

Asymptomatic children with *P. falciparum* gametocytemia ≥ 56 gametocytes/μL, parasitemia ≤ 1000 parasites/μL and negative for other *Plasmodium* species were selected as blood donors for the direct membrane feeding assay (DMFA) scheduled for the following day.

All children with confirmed malaria infection obtained treatment with the combination of artesunate (4 mg/kg body weight)/amodiaquine (10 mg/kg body weight), once daily for 3 days according to the guidelines of the National Malaria Control Programme. Children selected as gametocyte donors for the DMFA were treated the following morning after venous blood collection at the laboratory, those not recruited for the study were given the treatment the same evening by the village health workers.

### Anti-malarial plants and extracts

Neem (*A. indica* A. Juss., Meliaceae) and *G. senegalensis* J. F. Gmel (Combretaceae)*,* previously found to interfere with the sporogonic development of the rodent parasite *P. berghei* in *Anopheles stephensi* mosquitoes have been selected for this study [13,14]. The following plant parts and extracts were examined:

i) NeemAzal® (NA), a commercial methanol extract from neem seed kernels containing azadirachtin A 34%, other azadirachtins (azadirachtin B to K) 16%, salannins 4% and nimbins 2% (Trifolio-M GmbH, Lahnau, Germany).

ii) two extracts of neem leaves collected in the Oubritenga Province, Burkina Faso, namely: a) total EtOH extract (NLE); b) EtOAc phase of the EtOH extract (NLA) obtained by partitioning NLE between water and EtOAc. The leaves contained limonoids, with gedunin as a major member of this class, but were devoid of azadirachtin. After partitioning, limonoids were concentrated in NLA.

iii) EtOAc phase of the total ethanol extract of neem fruits collected in the Oubritenga Province, Burkina Faso (NFA). This plant fraction has been subjected to detailed phytochemical analysis and 10 triterpenoid derivatives have been identified. The most abundant limonoids were azadirone and azadiradione accounting for 70% of the total components. The gedunin content was estimated at 3%, azadirachtin was not present at detectable amounts [15].

iv) EtOAc extract of *G. senegalensis* stem galls, collected in the Bobo-Dioulasso area, Burkina Faso (GS).

Leaves and fruits of *A. indica* and *G. senegalensis* had been collected in the central region of Burkina Faso in June 2008. Plants were identified by Prof. Jeanne Millogo, professor of botanics at the Life Science Unit (University of Ouagadougou) and voucher specimen N°2 NFE (*A. indica*), N°1 GSE (*G. Senegalensis*) have been deposited in the Laboratory of Ecology at the University of Ouagadougou. Extracts were prepared and chemically characterized [13-15] at the Department of Pharmacy of the University of Naples “Federico II”.

All extracts were initially screened at the high dosage of 250 ppm to allow any possible transmission blocking effects to be detected even by compounds present at low concentrations. Extracts found active were then tested at decreasing doses.

### Transmission blocking experiments

The transmission blocking efficacy of the plant extracts on *P. falciparum* gametocyte field isolates was assessed on *An. coluzzii* mosquitoes, using a colony established in 2008 from field collected mosquitoes. For the membrane feeding assays, 4–5 day old females, kept without sucrose solution for 24 hours before the experimental infection, were used.

Approximately 8 mL of gametocytaemic blood was collected from each selected child by venous puncture using heparinized tubes. Care was taken to keep the blood tubes constantly at 37°C to avoid gametocyte activation leading to precocious gamete formation. Volumes of 10 – 35 μL of experimental extracts, dissolved in sterile distilled water for injection were added to 1 mL blood aliquots to obtain the desired final extract concentrations. Equal amounts of sterilized distilled water for injection were added to the control blood samples. Extract supplements and control blood mixtures were transferred to membrane feeders and 50 mosquito females per feeder were allowed to take blood for 30 to 60 minutes. Fed mosquitoes were separated from the unfed specimens and kept on a 10% sucrose diet. On day 7 after membrane feeding, midguts were dissected from all surviving females, stained with 1% mercurochrome in PBS (phosphate buffered saline, pH 7.2) and the presence and number of oocysts recorded for each mosquito. The transmission blocking activity of the plant extracts was estimated by determining both oocyst prevalence and oocyst density. For each test extract and concentration, two to five independent replicates were performed and prevalence and density values calculated for the treatment and control groups.

### Statistical analysis

Generalized linear mixed models were used to compare mosquito infection in control and treatment groups and estimate intervention efficacy [16]. Differences in oocyst prevalence (number of oocyst positive mosquitoes per group) were tested assuming the number of positive mosquitoes followed a binomial distribution. The number of oocysts per mosquito (including negatives mosquitoes) was best described using a zero-inflated negative binomial distribution. Ninety-five percent confidence interval estimates were generated using bootsrapping methodology.

### Ethical approval

The study was approved by the ethical committee of the Centre Muraz and filed under the registration number N/Ref. 003-2009/CE-CM. Parents or guardians provided written informed consent before children were enrolled into the blood collection protocol.

## Results

The transmission blocking activity of the four *A. indica* extracts and the *G. senegalensis* extract was investigated in a series of 31 membrane feeding experiments using *P. falciparum* gametocyte-positive blood from 18 different donors. Gametocyte densities varied among the experimental blood samples from 56 (threshold for inclusion) to 1760 sexual forms per microliter of blood; two thirds of the samples (12/18) displayed gametocyte densities between 96 and 416 (Table 1). Infection of control mosquitoes varied considerably between the experiments: oocyst prevalence ranged between 45% and 92% (mean 70%, CI_95_ 65.7-74.7) and oocyst densities between 1.4 and 151.6 (mean 31.5, CI_95_ 17.1-45.8; Tables 2 and 3).

**Table 1:** Blood samples used for membrane feeding assays

**Blood sample number**	**Hemoglobin (g/dL)**	***Plasmodium falciparum*** **(asexual forms) per microliter**	***Plasmodium falciparum*** **gametocytes (sexual forms) per microliter**
1	15.0	0	56
2	15.0	0	104
3	15.4	1040	120
4	15.2	0	128
5	13.4	400	216
6	15.1	0	528
7	14.8	0	112
8	15.2	560	96
9	12.2	800	64
10	15.6	400	232
11	14.7	0	400
12	14.0	0	1760
13	14	1440	64
14	12.1	80	416
15	12.9	880	344
16	13.5	480	184
17	14.1	2000	112
18	9.3	0	88

**Table 2:** **Transmission blocking activity of *Azadirachta indica *** **and *Guiera senegalensis *** **extracts at 250 ppm (initial screening dosage) on *Plasmodium falciparum *** **oocyst**

**Blood sample number**	**Gametocytes per μL blood**	Treatment^*^and concentration	**Prevalence of infected mosquitoes% (infected/total examined)**	**Number of oocysts/mosquito (CI**_**95**_**)**
1	56	control	53 (10/19)	1.79 (0.30-3.28)
		NA 250 ppm	0 (0/22)	0.00
		control	58 (15/26)	3.23 (1.34-5.12)
		NA 250 ppm	0 (0/15)	0.00
9	64	control	71 (39/55)	7.15 (5.10-9.19)
		NLA 250 ppm	20 (09/44)	0.32 (0.09-0.54)
13	64	control	54 (21/39)	1.95 (0.87-3.02)
		NLA 250 ppm	12 (05/42)	0.26 (0.00-0.52)
4	128	control	55 (18/33)	1.91 (0.91-2.91)
		NLA 250 ppm	35 (08/23)	0.57 (0.14-0.99)
10	232	control	76 (32/42)	15.69 (10.82-20.56)
		NLA 250 ppm	76 (13/22)	1.36 (0.54-2.19)
13	64	control	45 (15/33)	1.39 (0.59-2.20)
		NLE 250 ppm	42 (13/43)	2.67 (1.35-4.00)
5	216	control	53 (09/17)	18.88 (4.26-33.50)
		NLE 250 ppm	65 (15/23)	27.83 (10.20-39.45)
10	232	control	71 (35/49)	10.61 (7.16-14.06)
		NLE 250 ppm	84 (31/37)	20.92 (14.50-27.34)
18	88	control	56 (14/25)	2.28 (0.58-3.98)
		NFA 250 ppm	10 (03/30)	0.20 (0.00-0.45)
3	120	control	67 (32/48)	3.69 (2.28-5.09)
		NFA 250 ppm	41 (29/70)	1.84 (1.03-2.65)
14	416	control	66 (31/32)	63.59 (37.99-89.19)
		NFA 250 ppm	88 (30/34)	88.15 (60.08-116.21)
8	96	control	61 (25/41)	1.90 (1.11-2.70)
		GS 250 ppm	58 (21/36)	2.14 (1.05-3.23)
2	104	control	58 (21/36)	6.67 (3.74-9.59)
		GS 250 ppm	41 (09/22)	2.05 (0.63-3.46)
7	112	control	71 (27/38)	2.71 (1.55-3.87)
		GS 250 ppm	71 (36/51)	4.61 (3.14-6.07)

^*^NA: NeemAzal®; NLA: neem leaves EtOAc extract; NLE: neem leaves EtOH extract; NFA: neem fruits EtOAc extract; GS: *Guiera senegalensis* stem gall EtOAc extract.

**Table 3:** **Transmission blocking activity: dose dependent efficacy of NeemAzal® (NA) on *Plasmodium falciparum *** **oocyst prevalence and density**

**Blood sample number**	**Gametocytes per μL blood**	**Treatment and concentration**	**Prevalence of infected mosquitoes% (infected/total examined)**	**Mean number of oocysts/mosquito (CI**_**95**_**)**
11	400	control	64 (09/14)	51.36 (12.83-89.88)
		NA 70 ppm	0 (0/25)	0.00
12	1760	control	75 (12/16)	94.38 (38.29-150.46)
		NA 70 ppm	0 (00/25)	0.00
17	112	control	80 (12/15)	4.53 (2.69-6.38)
		NA 60 ppm	0 (0/31)	0.00
16	184	control	64 (29/45)	4.58 (2.96-6.19)
		NA 60 ppm	0 (0/56)	0.00
15	344	control	90 (09/10)	131.00 (49.69-212.31)
		NA 60 ppm	12 (03/25)	0.36 (0.00-0.83)
11	400	control	68 (17/25)	49.36 (22.92-75.80)
		NA 60 ppm	0 (0/25)	0.00
12	1760	control	75 (12/16)	94.38 (38.29-150.46)
		NA 60 ppm	0 (0/25)	0.00
17	112	control	85 (11/13)	3.92 (1.91-5.93)
		NA 50 ppm	23 (07/31)	0.35 (0.05-0.66)
16	184	control	59 (24/41)	2.68 (1.58-3.79)
		NA 50 ppm	0 (0/46)	0.00
15	344	control	92 (24/26)	151.58 (97.31-205.84)
		NA 50 ppm	36 (15/42)	2.29 (0.60-3.97)
11	400	control	82 (28/34)	66.50 (44.04-88.96)
		NA 50 ppm	0 (0/23)	0.00
6	528	control	90 (54/60)	42.85 (31.36-54.34)
		NA 50 ppm	18 (11/61)	0.30 (0.10-0.49)
17	112	control	67 (18/27)	6.56 (3.60-9.51)
		NA 20 ppm	52 (12/23)	2.57 (0.86-4.27)
16	184	control	84 (38/45)	4.42 (2.92-5.93)
		NA 20 ppm	41 (26/63)	0.95 (0.56-1.34)
15	344	control	81 (29/36)	92.69 (47.92-137.46)
		NA 20 ppm	60 (12/20)	44.85 (7.90-81.80)
6	528	control	85 (44/52)	32.90 (22.36-43.45)
		NA 20 ppm	88 (44/50)	27.24 (19.12-35.36)

**Figure 1:**
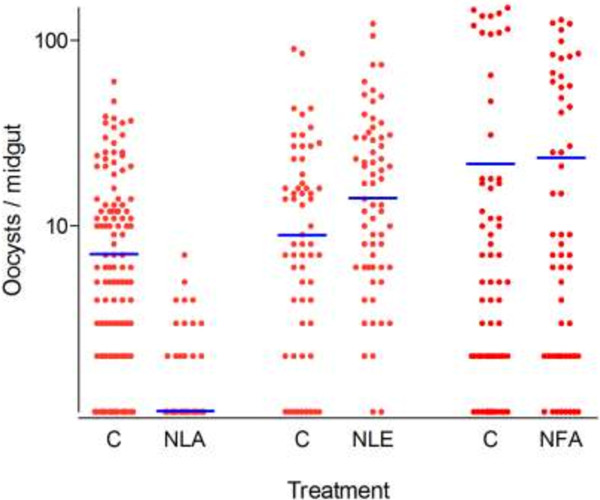
**Effect of 250 ppm Neem extracts on oocyst formation of *P. falciparum *field isolates in *An. coluzzi *mosquitoes.** NLA (leaves EtOAc extract, 4 independent replicates), NLE (leaves EtOH extract, 3 independent replicates) and NFA (fruits EtOAc extract, 3 independent replicates), C (controls). Each point represents the number of oocysts per mosquito (including mosquitoes with zero oocysts); horizontal bars (blue lines) indicate geometric means calculated on all mosquitoes (negatives included).

Initial screening of the plant extracts at 250 ppm revealed transmission blocking activity in two out of the four *A. indica* preparations (Table 2). At this high dose, the commercial seed kernel extract NeemAzal® completely blocked transmission. The total ethanol extract from the neem leaves did not exhibit any activity (Table 2, Figure 1), while the EtOAc phase of this extract reduced oocyst prevalence by 59.0% (CI_95_ 12.0 - 79.0; *p* < 10^-4^) and oocyst density by 90.5% (CI_95_ 86.0 - 93.5; *p* < 10^-4^). The mean percentage inhibition for each experiment compared to its control from the four independent replicates was respectively 96.0% (CI_95_ 94.0 - 98.0), 87.0% (CI_95_ 83.0 - 99.6), 70.0% (CI_95_ 66.0 - 85.0) and 91.0% (CI_95_ 89.0 - 95.0). The neem fruit EtOAc extract showed inconsistent results: in two experiments using blood with relatively low gametocytemia (120 and 88 gametocytes/μL) oocyst prevalence and density were reduced by 58.54% (CI_95_ 22.69 - 90.38, *p* = 0.007) and 65.83% (CI_95_ 63.98-65.82, *p* = 0.008) in treated mosquitoes compared to controls. The third replicate experiment however, performed with a blood sample rich in gametocytes (416 gametocytes/μL) yielded numerous oocysts in both, treated and control mosquitoes (Table 2). No evidence of transmission blocking activity was found with the *G. senegalensis* EtOAc extract from stem galls.

**Figure 2:**
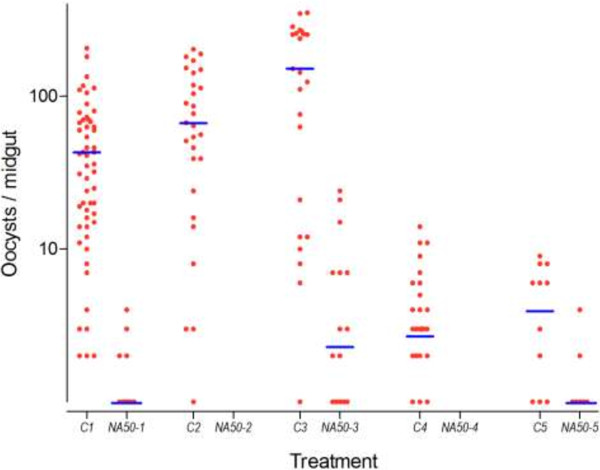
**Effect of 50 ppm NeemAzal® on sporogonic development to oocyst formation (5 independent replicates).** Control groups: C1- C5, treatment groups: NA50-1 to NA50-5. Each point represents the number of oocysts per mosquito (including mosquitoes with zero oocysts); horizontal bars (blue lines) indicate geometric means calculated from all mosquitoes (negatives included).

**Figure 3:**
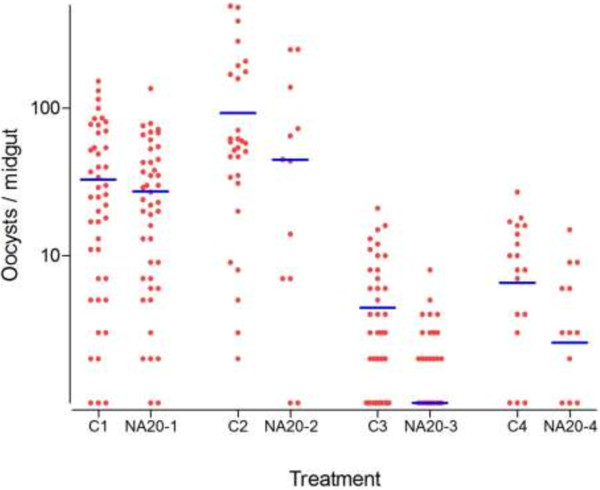
**Effect of 20 ppm NeemAzal® on sporogonic development of oocyst formation (4 independent replicates).** Control groups: C1- C4, treatment groups: NA20-1 – NA20-4. Each point represents the number of oocysts per mosquito (including mosquitoes with zero oocysts); horizontal bars (blue lines) indicate geometric means calculated from all mosquitoes (negatives included).

When NeemAzal® was tested at decreasing dosages it completely blocked mosquito infection at 70 ppm in two independent replicates, at 60 ppm in four out of five, and at 50 ppm in two out of five replicate experiments (Table 3). At 50 ppm NeemAzal® treatment (5 replicates), a strong impact on both oocyst prevalence and density was observed: 15.4% (CI_95_ 0.00-34.67) of the treatment group were found positive for oocyst as compared to 81.6% (CI_95_ 65.16-98.04) of control mosquitoes, corresponding to a reduction of 79.9% (CI_95_ 58.72 - 81.29; *p* < 10^-6^) (Figure 2). On NeemAzal®-treated mosquito midguts a mean of 0.62 (CI_95_ 0.25 - 0.98) oocysts was recorded, 99.06% (CI_95_ 98.52 - 99.40; *p* < 10^-6^) less than on control midguts (51.34, CI_95_ 39.39 - 63.30; Table 3, Figure 2). At 20 ppm, oocyst-positive mosquitoes were observed in each of the 4 treatment group replicates and the mean oocyst prevalence was not different in the NeemAzal® (60.25%, CI_95_ 28.31 - 92.19) group with respect to the control groups (79.25%, CI_95_ 65.98 - 92.52; p = 0.17). However, analysis of oocyst densities still evidenced a reduction of 52.69% (CI_95_ 34.81 - 65.67; *p* < 10^-6^; Table 3 and Figure 3).

## Discussion

Extracts from *A. indica* (neem) were found to contain components with transmission blocking activity against *P. falciparum* field isolates in our vector infection experiments conducted in Burkina Faso. Of the different *A. indica* extracts examined, NeemAzal®, an azadirachtin-enriched preparation of neem seeds showed prominent inhibitory activity on parasite development in the vector. When added at a concentration of 60 ppm to gametocytaemic blood from *P. falciparum* infected donors and membrane fed to *An. coluzzii* females, oocyst development was completely suppressed in four out of five independent replicates. At 50 ppm, oocyst prevalence was reduced by 80% and density by 99% and at 20 ppm, a 53% decrease in oocyst density was still observed.

Despite considerable variation of gametocyte density between replicates, consistent dose dependent results were obtained, substantiating the specificity of the NeemAzal® action and providing support for the robustness of the *P. falciparum* field isolate membrane feeding assay developed at IRSS in Bobo-Dioulasso. These results establish the transmission blocking activity of NeemAzal® against the human parasite *P. falciparum*, and are in excellent agreement with the previously reported activity in the murine parasite model *P. berghei* and *An. stephensi* mosquitoes [14].

The neem tree (*A. indica*) is a popular medicinal plant used in various Asian and African countries for the cure of various ailments and illnesses caused by infectious agents, including malaria. More frequently leaves, but also fruits, seeds and the bark are employed for the preparation of traditional remedies [13-17]. The efficacy of these preparations has been associated with the large number of secondary metabolites present in the plant, including at least 50 bioactive limonoids [18]. Studies aimed at detecting the molecules responsible for the antimalarial activity of neem extracts found that the limonoids nimbolide and gedunin displayed major inhibitory effects on *in vitro* growth of *P. falciparum* asexual blood stages [19-22]. Azadirachtin, moderately active on asexual blood stages [23], has been demonstrated to inhibit the exflagellation process of *P. falciparum* and *P. berghei* microgametocytes *in vitro*, the initial phase of sporogonic development in the vector [24,25]. In studies conducted with the azadirachtin-enriched standardized extract NeemAzal®, the product was found to completely block the development of *P. berghei* in *An. stephensi* mosquitoes when fed on gametocytemic mice previously treated with NeemAzal® at an azadirachtin concentration of 50 mg/kg body weight [14]. The reduced number of ookinetes recorded in the NeemAzal® treated group suggested an interference with the parasite development already before zygote formation and provides indirect evidence of azadirachtin interference with microgametogenesis *in vivo*. In addition, post-zygotic forms from NeemAzal® treated mosquitoes displayed evident morphological alterations and no mature ookinetes could be detected at the 50 mg/kg dosage, indicating that NeemAzal® action is targeted to the early sporogonic stages developing in the midgut lumen. This assumption was further supported by the observation that NeemAzal®, when blood fed to already infected mosquitoes did not interfere with oocyst development [14].

Several studies report that azadirachtin and other limonoids present in neem extracts are active on mosquitoes [26,27]. Exploring the insecticidal activity of NeemAzal® in *An. stephensi,* it emerged that females membrane fed with NeemAzal® containing bloodmeals at an azadirachtin concentration of 100 μg/mL displayed reduced blood feeding capability and produced fewer eggs [28]. This reduction in oviposition was mirrored by degenerative tissue damage in ovarian follicles [28]. In addition, studies in laboratory animals indicated a very low acute toxicity of NeemAzal® by all routes tested; lethal dose for 50% of the animal population (LD_50_) was higher to 5000 mg/kg body weight in rat [29].

In this study, a transmission blocking activity on *P. falciparum* field isolates was also displayed by the EtOAc fraction from neem leaves (NLA). Added at 250 ppm to blood from gametocytaemic donors and membrane fed to *An. coluzzii* mosquitoes, NLA reduced oocyst prevalence by 59% and oocyst intensity by 90%. Since this extract does not contain detectable levels of azadirachtin, this activity must be attributed to the presence of other neem components. Gedunin, which is abundant in NLA might be involved. This molecule, previously reported to possess gametocytocidal activity *in vitro*[30], certainly deserves attention for further studies. Comparison of the results obtained with the total neem leaf ethanol extract and its EtOAc fraction (NLA, no evidence of transmission blocking activity in the first and in the second replicate), allows us to assume that the unidentified active molecules have apolar characteristics. This feature, together with the abundance of limonoids in NLA suggests their belonging to the limonoid class.

The examined EtOAc extract from neem fruits did not show consistent transmission blocking inhibitory effects at 250 ppm initial screening dose. Detailed phytochemical analysis of this plant fraction allowed the isolation of 10 triterpenoid derivatives including the identification of two new limonoid molecules named neemfruitins A and B [15]. Since the major components of this the extract were identified as azadirone and azadiradione (70% of the total components), a transmission blocking activity can be, most likely, excluded for these two molecules. Azadirachtin was not present in detectable amounts and gedunin accounted for only 3% of the total neem fruit limonoids [15] and could potentially be responsible for the partial activity displayed. Interestingly, *in vitro* screening for anti-blood stage activity using a *P. falciparum* chloroquine sensitive (D10) and resistant (W2) strain revealed prominent *in vitro* schizontocidal activity of the EtOAc neem fruit extract. IC_50_ values of 1.31- 3.35 μg/mL were found. Similar values were observed for the isolated molecules azadirone, gedunin and neemfruitin A [15]. Given the relatively low abundance of highly active limonoids in these extracts, their prominent activity cannot be explained only on the basis of additive effects, but a synergistic action between the constituent molecules should be taken into account [15]. In addition, the *in vivo* activity of neem fruits has been demonstrated: mice treated at a daily oral dosage of 200 mg/kg crude extract over 9 days and exposed to infectious mosquito bites on day 3 of treatment displayed parasitaemia levels reduced by 45% [17].

Stem gall ethyl acetate extract from *G. senegalensis*, the second anti-malarial plant selected for this study, did not display transmission blocking activity. It has been included in this study since remedies based on the galls of this plant are frequently used in the Bobo-Dioulasso area for the treatment of fevers and similarly to neem, *G. senegalensis* contains abundant terpenoids and phenolic compounds (tannins, flavonoids) [31].

## Conclusion

In conclusion, this study confirmed activity of limonoid-rich medicinal plant extracts on the sporogonic development of *P. falciparum* isolates in *An. coluzzii* mosquitoes. The results strongly suggest azadirachtin to be an important compound, but the results also provide evidence for the presence of at least one other molecule with transmission blocking activity. Screening many purified molecules would have not been practical in this study because of logistical and ethical limitations (e.g. distance from the field to the lab; amount of blood that can be collected from individual donors, especially children). However, by focusing on a small panel of extracts with different limonoid content profiles it was possible to deduce useful information on the transmission blocking activity of single constituent molecules.

Considering the anti-plasmodial activity of neem limonoids on different life cycle stages of the parasite these compounds hold promise for the design of new, effective, multi-stage combination medicines. As an example, herbal medicines designed as preventive-transmission blocking formulations, if used by entire communities, may reduce incidence of malaria cases and decrease the intensity of transmission. Studies aimed at assessing bioavailability of pure azadirachtin and azadirachtin rich preparations have been initiated in order to validate the feasibility of the approach.

## Competing interests

I declare that no competing interests existed for the authors or the institute before, during and after preparing and submitting this paper for review.

## Authors’ contributions

RSY participated in study design, carried out the experiments, performed the statistical analysis, and drafted the manuscript; LL participated in study design; RKO, DFA, FAY and KBY participated in the execution of the experiments; TSC: conducted the statistical analysis; RSY, GL and OTS participated and helped with plant extraction and partitioning; LCG, AC and GKC participated in study design and helped with manuscript revision; JBO and AH coordinated the work, participated in study design and critically revised the manuscript. All the authors read and approved the final manuscript.
